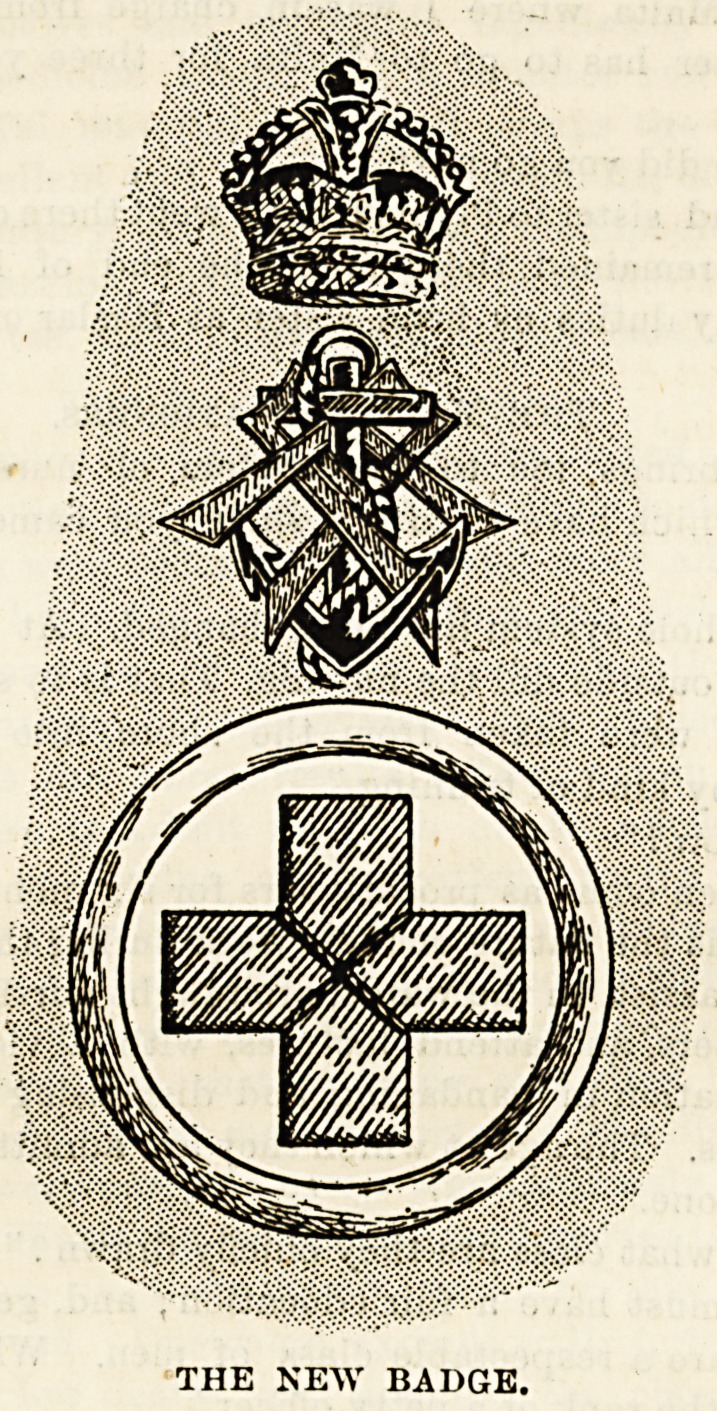# The Hospital. Nursing Section

**Published:** 1902-05-24

**Authors:** 


### The Hospital.

### Nursing Section.

Contributions for this Section of "The Hospital" should be addressed to the Editor, "The Hospital" Nursing Section, 28 & 29 Southampton Street, Strand, London, W.C.

No. 817.—Vol. XXXII. *SATURDAY, MAY* 24, 1902.

### Notes on News from the Nursing World.

### NURSING IN THE NAVY.

It will be seen from the report of our commis-missioner's visit to the Royal Naval Hospital at Haslar that the work of the members of the Naval Service is distinctly arduous. The hours of duty are not the same at all the hospitals, and possibly the obligations of the sisters at Haslar are unusual. But for one woman to have charge of four or five wards containing 14 beds in each, with a certain proportion of bad cases, during the day, and of half the hospital during the night, is no light matter. It is true that each sister has the assistance in the day of two or three young male probationers and an attendant. But the former are only undergoing their six months' training, and the attendants are probationers promoted at the end of the six months. The point is that practically, as the head sister says, the sisters do the whole of the actual nursing. The head sister does not suggest that they find the work too laborious, though she says that they cannot always be spared when their time for a Sunday, or a half-day, off duty comes. The alterations in the service which are expected as the result of the Queen's assumption of the presidency will doubtless include, among other things, the appointment of more sisters. This, at any rate, seems to us to be required in the interests both of patients and of nurses.

### THE LATE MATRON OF BELLEVUE HOSPITAL, NEW YORK.

The resignation of Miss Agnes S. Brennan, superintendent and matron of Bellevue Hospital, New York, has naturally excited a great deal of attention in the American nursing world. Although the event was, generally speaking, quite unexpected, it has since transpired that Miss Brennan had for some time determined to resign on May 1st when she completed two decades of service in one of the greatest of hospitals on the other side of the Atlantic. In addition to her duties as matron of the Bellevue Hospital, and superintendent of the training school, she has also been head of the Bellevue Registry for Nurses. Some idea of her devotion to her onerous work may be gathered from the fact that for seven years the only summer holiday she took was a trip to Buffalo to attend the Congress last year. To her efforts the organisation of the Bellevue Alumnæ Association was largely due, and it was her forethought that secured for the Bellevue graduates a private pavilion and provision for their ease when sickness overtakes them. Throughout her career Miss Brennan steadily addressed herself to the task of turning out faithful nurses, and there is no doubt that some of the best in the United States were trained under her auspices.

### PRIVATE NURSING IN RANGOON.

As we anticipated, there is a desire on the part of many [nurses to make a rush to Rangoon. Instead of replying individually to the number of correspondents who have written to us for further details than were given in these columns on the 10th of May, we answer them collectively here, as far as we are able. Those who feel that they can face the climate and other drawbacks might apply to the Colonial Nursing Association, Imperial Institute, S.W. There is a nursing institution at Rangoon, but we do not know how long it has been established. The correspondent to whom we are indebted for the information that nurses are wanted states that September and October are the best months for going out. She suggests that a nurse desiring to proceed to Rangoon might, by advertising about that time as companion to a lady, or as willing to take the charge of an invalid, obtain her passage for nothing. She also thinks that a nurse might act as stewardess on one of the out-going steamers which start respectively from the London Docks and from Liverpool, although such posts are not easily obtainable. These are matters which nurses must decide for themselves; but we emphatically warn our readers not to take risks which they are unable to afford, or by want of prudence to expose themselves to disappointment, annoyance, and perhaps pecuniary loss.

### SERIOUS ALLEGATIONS AGAINST AN ISOLATION HOSPITAL.

The allegations of a thoroughly well-informed correspondent respecting the management of the Whitby Isolation Hospital require immediate attention, unless the authorities desire the perpetual presence of diphtheria in the town. Our correspondent, it will be observed, states that there are no sanitary arrangements whatever in the Isolation Hospital; that the contents of bed-pans are thrown into an open hole in a field 15 or 20 yards from the ward; that, in the absence of a bath-room, a patient who is leaving has a tub in the ward, is bathed and dressed there and sent straight home; that the only means of disinfecting clothes is in an old cab; that the nurses have no room of any kind; and that they either sleep in the diphtheria ward or in a small passage near the kitchen. The matron's husband is the porter, and the presumption is that she is not trained. However that may be, the condition of things thus described is not only a reproach to whoever is responsible for it, but a menace to the public health.

### A DISTRICT NURSE FOR OBER-AMMERGAU.

It was originally proposed that the sum of a thousand pounds should be raised to endow two beds at the Ober-Ammergau Cottage Hospital as a tribute from the English visitors to the Passion Play. This proposal has, however, been abandoned for one intended to secure a salary to provide a second nursing sister. The Burgermeister and his full council have received the alternative proposal with cordial and grateful satisfaction. The chief duty of the second sister will be to go out and nurse the sick in their own homes. She will, in fact, be the district nurse of Ober-Ammergau. Now that it has been decided to confer what we cannot doubt will be the greater boon to the people, we trust that the remainder of the sum still required will soon be made up by the numerous well-to-do persons who are in the habit of visiting Ober-Ammergau and deriving pleasure and profit from the performance of the Passion Play.

### A GOOD START AT DARLASTON.

If the Darlaston District Nursing Institute continues to receive the support which it has obtained in the first year of its existence, there will be no occasion for anxiety as to the financial position. The income amounted to £331, as against an expenditure of £227. Moreover, owing to initial outlay in the provision of nursing appliances, the expenditure was heavier than it is expected to be in the future. A most pleasing feature of the receipts was that the working classes contributed £109, or a third of the whole. They have set an example which we should like to see widely followed. As to the work of the nurses, upwards of 500 cases were attended, and upwards of 9,000 visits were paid—a conclusive proof that the Institute was urgently needed. In the summer and autumn of last year Darlaston was the scene of an epidemic of typhoid, and the devotion of the nurses greatly impressed the residents.

### VIEW DAY AT ST. BARTHOLOMEW'S HOSPITAL.

The hospital of St. Bartholomew held its annual court of almoners and visitors last week. The day was not auspicious: rain fell at intervals, and the gusts of wind were trying, waving as they did the matron's cap ends like a pennon behind her. An animated crowd of relatives and visitors thronged the quadrangle, and shortly before three the procession started on its round of the 28 wards. The matron led, preceded by the silver mace, and closely followed by Sir Trevor Lawrence, the treasurer, and the rest of the party. They were received in each ward by the sisters and medical staff; a short conversation followed, the procession reformed and proceeded to the next ward. The business of the day being over, tea and talk ensued, and many were the exclamations of admiration at the exquisite decorations. "President," a women's surgical ward, was a sight to remember, pink and white roses being lavishly used and beautifully mirrored; the mantel, with its draping of pink silk and green foliage, was very effective. "Paget" was gorgeous, so were "Kenton" and "Hope." "Elizabeth" had favoured white, and looked very chaste. Pink and primrose were the predominating colours; lilac was only seen in one ward, but it was not sufficiently in bloom to be pretty. One small patient in "Kenton" caused much amusement to the guests. He sang very sweetly nearly the whole of the afternoon, but about 5.30 he said he felt tired, and he rested his little dark head lovingly against Sister's cheek, waiting for "cot" time. Many old nurses were present, and one lovely bride was talking now about "my husband" as formerly she did about "my ward."

### SIX MONTHS' PROBATION FOR A SUPERINTENDENT NURSE.

A curious feature of the election of superintendent staff nurse at the South Charitable Infirmary and County Hospital, Cork, was that the committee who recommended the choice of Miss Walsh proposed that she should be appointed "on probation for three months." This was subsequently altered to six months. Seeing that the credentials of Miss Walsh were regarded as entirely satisfactory, it is not easy to understand why a temporary appointment should have been made. No objection was offered by the successful applicant for the position, but anything in the nature of a system of appointing superintendent nurses on probation is not desirable.

### MEMORIAL TO AN ARMY NURSE.

On Thursday evening last week at a special service held at St. John's Church, Tue Brook, Liverpool, the ceremony took place of unveiling a memorial to the memory of Nursing Sister Elizabeth Stuart Jones, of that parish, whose death from enteric fever in South Africa took place two years ago. The service, which was attended by the relatives and friends of the deceased, consisted of the reciting of the office for All Souls' Eve, and in the course of an address the Vicar referred to the self-sacrifice and devotion to duty which throughout had characterised the life of Sister Jones. It will be remembered that, during her nursing career, she was connected with the Preston, Dorset, Warrington, and Cardiff Infirmaries, and at the time of sailing to the seat of war held the post of matron at St. John's Hospital for Diseases of the Skin, Uxbridge Road, London. The tablet, which consists of brass on a black marble base, bears the following inscription:—"To the Glory of God and in Memory of Nursing Sister Elizabeth Stuart Jones, of this parish, who while voluntarily fulfilling her duties tending the sick and wounded (nobly forgetful of her own safety) was stricken with fever and passed away in the Military Hospital, Bloemfontein, South Africa, May 16th, 1900, aged 29. Her body lies resting in the cemetery there."

### THE DUTY OF BOURNEMOUTH.

There is an impression that Bournemouth is one of the few towns in which there are no poor. Possibly the low rates and the general appearance of prosperity are the cause of this idea, which is none the less a delusion. We must, however, confess to surprise that with so many wealthy and well-to-do residents Bournemouth should not even provide sufficient money to cover the moderate expenditure of the District Nursing Society. That there are poor in the beautiful garden town is attested by the fact that the nurses attended 376 cases and paid more than 16,000 visits. Moreover, this is a large increase on past years. The receipts for 1901 amounted to £399 17s. 6d. and the disbursements to £406 3s. 10d. The balance on the wrong side is small, but the point is that there ought to be a substantial balance on the right side. It further detracts from the limited generosity of the Bournemouth people that £62 17s. of the receipts represented fees paid by patients themselves. The most rising of all watering-places should find it easy to contribute £500 a year for its sick and suffering poor.

### A BAPTISMAL CERTIFICATE DEMANDED.

The Armagh Board of Guardians have again selected a nurse for the fever hospital, the voting for the two selected candidates being, as on former occasions, influenced by considerations of religion. The nurse who "is believed to be a Protestant" obtained twenty votes as against six given to the nurse who avowed herself a Roman Catholic. We regret that the majority of the Armagh Guardians should persist in raising an issue which ought not to be raised, but we do not think that the minority were justified in demanding that the successful candidate should produce her "baptismal certificate." It is reasonable and necessary that the production of the certificate of training should be asked for; but not a certificate of baptism.

### "SHOALS" OF APPLICATIONS AT NEWTON ABBOT.

The inquiry at Newton Abbot Workhouse into the alleged interference on the part of Dr. Ley, a member of the Board of Guardians, has taken place. Dr. Culross, who recently resigned the position of medical officer, offered no personal testimony to substantiate his allegation of interference with himself, although he complained that Dr. Ley had put questions to patients as a medical man and had thrown doubt on his diagnosis. Miss Jeffery, who was superintendent nurse at Newton Abbot for nearly four years, said that Dr. Ley's questions would often annoy because they were questions which ought only to be asked by the medical officer, but she also stated that Dr. Ley did not interfere with her work, and added, "I might be ruffled a bit, but I could go ahead." Miss Fisher, who until recently was superintendent nurse, alleged that Dr. Ley weakened her authority with the assistant nurses, and very often questioned her in the capacity of a medical man. After other evidence Dr. Ley, who denied interference, said he had devoted his life to looking after the poor in the workhouse, and he was going to continue to do so. He had supported Dr. Culross in every way, and Superintendent Nurse Fisher was perfectly friendly with him until four nurses resigned. The chairman pointed out that for 7½ years Dr. Culross never complained of interference and made no sign until the medical officer and nurses fell out. He suggested that Dr. Culross should withdraw his allegations and that Dr. Ley should assert that he never intentionally interfered. Dr. Culross, however, declined, and as the result of a lengthy inquiry, the Board, by 32 votes to 8, declared that no intentional interference had taken place. We hope that the bickerings at the workhouse will now cease, and that there will soon be a good permanent nursing staff, the persistent advertising having at last been rewarded with success. During the inquiry it was incidentally mentioned that there were "shoals" of applications.

### A REASONABLE BOARD OF GUARDIANS.

The Ecclesall Board of Guardians have shown themselves amenable to reason. The superintendent nurse applied for an increase in her staff. The request was modest enough; she only asked for one more probationer. The Guardians at first manifested a disinclination to assent even to this addition. However, the medical officer expressed his opinion that the superintendent nurse had made out a case, and she herself explained the urgency of the need. To these representations from the two persons who are most competent to judge of the position, the Guardians eventually wisely yielded.

### A CORONER'S FREAK.

A remarkable incident is reported from Bootle. At an inquest on the death of a woman in Bootle Hospital, the coroner for South-west Lancashire summoned a nurse belonging to the staff to give evidence concerning the death of the patient, and ignored the medical officer. It does not even seem to have been necessary to summon the nurse as an additional witness; and if it had been, the medical officer, who is, of course, the person responsible in such a case, should certainly have been summoned also. The nurse was obliged to obey the summons, but the coroner had no right to place her in such an invidious position.

### A CLOSED CONTROVERSY.

Sir, my gratitude profound,
I accord you on the ground,
That you've done what every proper nurse would wish you:
Bidding friends with pens profuse,
A fresh subject introduce
In your next forthcoming interesting issue.

I'm a thankless nurse, no doubt,
For I really can't make out,
Where "Medicus" and Co. are wildly driving
By stirring up this strife:
Exposing hardships in our life
Which we nurses (as a rule) to hide are striving.

In her patience under trial,
In her gracious self-denial,
In her modesty of thought and word and bearing;
Lies the nurse's greatest strength,
Not in setting forth at length
To the public—all her wrongs, in language glaring!

Patients at our hands deserve,
The fair flower of sweet reserve;
And to them and self a nurse's sacred duty
Is the silence of the tomb
On all that happens in each room:
For in reticence there's safety, honour, beauty!

As the controversy's ended,
And what's said cannot be mended,
I will trespass, Sir, no longer on your time;
Lest you write me down a bore,
I will poetize no more,
But close, without delay, my little rhyme.—*J. De R.*

### SHORT ITEMS.

The *Sicilia* arrived from South Africa last week with A. N. Martin, A.N.S.R., and F. Stonham, Civil Nurse, on board. The former has been granted one month's leave and is going to resign; the latter requires three months' leave and returns to South Africa.—The following Nursing Sisters arrived at Southampton from South Africa on the 14th inst. on board the *Orotava*: J. E. Smith (no leave required), B. M. Cornell (one month's leave), G. Westbrook (one month's leave), all members of the A.N.S.R.; E. M. Bernhard-Smith, Victorian N.S. (two months' leave required).—The Clothworkers' Company have contributed £25 to the East London Nursing Society. This is their eighth donation, and brings the company's total grants up to £225.

### Lectures on Gynæcology for Nurses.

By Robert Jardine, M.D., M.R.C.S., F.F.P. and S.G., F.R.S.E., Senior Physician to the Glasgow Maternity Hospital, Examiner in Midwifery to the University of Glasgow.

### XII.—PELVIC PERITONITIS.

Perimetritis is another name for this, but we shall use the term peritonitis to prevent confusion with parametritis or cellulitis. It is an inflammation of the peritoneal lining of the pelvis. It is one of the commonest inflammatory diseases of this region. It is much commoner than pelvic cellulitis, and like it is an infective process, *i.e.*, it is due to the absorption of micro-organisms or of their products. Formerly injuries and colds used to be looked upon as causes, but now it is pretty generally admitted that these will not cause it if sepsis be not present. After exposure to cold or an injury an attack often occurs, but sepsis has been present at the time, and the lowering of the vitality of the tissues by the cold or injury has given the micro-organisms their chance to invade tissues which under ordinary conditions could resist their onslaught. There is, as it were, a constant warfare going on between the tissues of the body and micro-organisms, and we may look upon pelvic peritonitis as an attempt on the part of the pelvic tissues to confine the ravages of the invading host to the pelvis and to prevent them working havoc throughout the general peritoneal cavity. Adhesions quickly form and shut off the pelvic cavity from the general peritoneal cavity. This is effectual in many cases, and we have a localised inflammation which gradually subsides; but if the invasion has been a very virulent one, as of streptococci after parturition, there may be no time for the formation of adhesions; or if these have formed they may not be able to stem the onrush of invaders, and the result is extension to the general peritoneal cavity and violent general peritonitis, which will probably be fatal.

Pelvic peritonitis may then be said to be the result of septic absorption. In puerperal sepsis it is always more or less present, and in bad cases the inflammation is unfortunately not confined to the pelvis. In other cases it is generally a result or complication of some pre-existing pelvic disease. In the vast majority of cases the infection has spread from the Fallopian tubes.

The Fallopian tubes extend outwards from the upper angles of the uterus on either side between the folds of the broad ligaments. Each tube curves round towards the ovary of its side and ends in a trumpet-shaped extremity by a number of finger-like projections called fimbriæ. One of these is attached to the ovary. Where the tube pierces the uterine wall the calibre will only admit a bristle, but at the outer end it will admit a quill. The cavity of the tube is continuous with that of the uterus, and that of the uterus with the vagina through the cervical canal, so that we have a continuous canal from the outside of the body into the peritoneal cavity. You must not imagine that there is a wide open passage between the air and the peritoneal cavity. As a matter of fact the vagina is not a gaping passage, as many diagrams would lead you to suppose. Its walls lie together, and in the uterus there is no open cavity, but by the passage of instruments one can soon form an open canal. Fluid can be forced right into the peritoneal cavity through the tubes, and for that reason intrauterine injections should never be given unless there is an arrangement to allow of a very free return of the fluid. For that reason a double-channelled nozzle is generally used. Now if a woman has a septic inflammation in the vagina, generally gonorrhœal, and nothing is done to cure it, it will gradually spread upwards through the cervical and the uterine canal and then into the tubes. The mucous lining is continuous, and there is nothing to stop the gradual invasion unless the tissues can overpower the organisms, and this they are unfortunately not able to do where gonococci are concerned. The effect of gonococci on a mucous membrane is that pus is quickly formed in large quantities. You will notice this in a case of ophthalmia in a new-born child. At first there is a copious watery secretion followed in a few hours by large quantities of pus. Gonococci are the cause here. Now if you have the same going on in the vagina and uterus the woman does not suffer very much except from the inconvenience of a profuse vaginal discharge, as there is free drainage. It is a different matter when the tubes are affected. At the best the minute opening of the uterine end of the tube would not admit of free drainage, and the inflammation will have completely closed it. The result is that the tube becomes distended with pus, and the outer end, which opens directly into the peritoneal cavity and is fairly wide, will allow of an escape of pus which will set up a localised peritonitis. The adhesions which will form may fortunately close the end of the tube, and the pus thus be prevented from getting into the peritoneal cavity; but this does not remove all the danger, as ulceration of the tube may take place, or the ovary may become affected from the tube, and this be a source of infection to the peritoneum. If there should be any cystic disease of the ovary suppuration is very likely to occur in the cyst. A patient who has pus in her tubes is sure to have repeated attacks of pelvic peritonitis from gradual leakage of the pus.

Another cause of peritonitis is the presence of a new growth in the pelvis. With ovarian tumours this is common. If you have been present at abdominal sections you will have noticed the surgeons often have considerable difficulty in dealing with what they call adhesions. These are the results of former attacks of peritonitis.

Pelvic peritonitis is found in connection with pelvic hæmatoceles, *i.e.*, with collections of blood in the pelvis. In most cases the blood comes from a ruptured tubal pregnancy or from a tubal abortion. Fortunately the peritonitis quickly shuts off the pelvic cavity from the general one, and in this way the blood becomes roofed over. This prevents an excessive loss of blood and may save the patient's life.

In appendicitis the inflammation may spread to the pelvis, and again in pelvic cellulitis the peritoneum is liable to be affected, as it lies in close contact with the inflamed cellular tissue.

*Appearance of the Inflamed Parts.*—The normal peritoneum is a smooth glistening membrane. When inflammation attacks it it loses its glistening appearance and becomes dull and slightly rough. The vessels become engorged with blood. Lymph is poured out on the surface and adhesions rapidly form. There is also effusion of serum, and this naturally gravitates to the lowest part of the pelvis, viz., into Douglas' pouch, or into any space formed by adhesions. The lymph which is thrown out coagulates and makes the pelvis feel through the vagina as if plaster of Paris had been poured into it and allowed to set.

The inflammation may be entirely confined to the pelvis, but it sometimes extends to the general peritoneum. If it has been confined to the pelvis gradual recovery will take place by absorption, but serious adhesions will be left behind. The tubes and ovaries may be bound down and the uterus may be forcibly drawn out of its normal position by the contraction of adhesions. In some cases suppuration may occur, especially when suppurative disease is the cause, and a pelvic abscess may form just as with cellulitis. It may open in the same way, and if it opens internally it will set up general suppurative peritonitis and cause death.

### LECTURES ON GYNÆCOLOGY FOR NURSES.—*Continued.*

*Signs and Symptoms.*—The patient complains of great pain in the lower part of the abdomen, and her pulse and temperature have risen. Rigors may occur. Vomiting may be very troublesome. The abdomen may be distended generally or it may be only locally. The bowels will be constipated, the tongue furred, and headache may be present. The patient will lie with both legs drawn up and her abdominal muscles fixed There will extreme tenderness over the part. When the bowels move she will suffer considerable pain, and micturition may also be painful. There may be retention of urine.

After the acute stage passes off the pain will lessen, but movement will cause pain, and if the patient is up going about she will complain of great pain in the back.

At first a vaginal examination will only reveal great tenderness and heat in the parts, with some fulness and fixation, but as soon as the effused lymph is organised the pelvis will feel as if it had been filled with plaster of Paris from above and this had set. You may find an indistinct fulness high up in the pelvis. This is from serous effusion. Or there may be a distinct bulging tumour behind the uterus from encysted serous fluid. A bimanual examination will not reveal much unless the patient is anæsthetised, as the abdominal muscles are too tense.

The chronic form often follows the acute, but it frequently develops slowly of itself.

*Signs and Symptoms.*—The patient complains of backache, profuse leucorrhœa, increased menstruation, and sterility. A vaginal examination will cause great pain, and will reveal obscure thickening in the fornices. The uterus will generally be displaced and more or less bound down by adhesions.

### Queen Alexandra's Naval Nursing Service.

### A VISIT TO HASLAR: BY OUR COMMISSIONER.

Since it was announced in February that Her Majesty, Queen Alexandra, had consented to become President of the Naval Nursing Service, and thus to identify herself as prominently with it as with the Army Nursing Service, there have been many inquiries from readers of The Hospital for information respecting the conditions on which it is possible to become a Naval Sister, and also respecting the nature of the duties which those who join are required to perform. On the principle that it is best to apply to the place where the practical work is carried on, I asked Miss Mackay, the Head Sister at the Royal Naval Hospital, to see me, and, receiving a courteous letter of acquiescence, I paid a visit to Haslar the other day. The town of Gosport is dominated by the huge building in which the temporarily disabled Jack Tar can always find shelter and succour. Its usefulness cannot be denied, but to architectual beauty it makes no pretensions, and the new quarters of the medical staff and of the sisters, which are quite on modern lines, are in striking contrast with the barrack-like appearance of the hospital. To traverse the whole of the wards is almost a pilgrimage, and time did not allow me to make the attempt; but the head sister was good enough to show me sufficient to enable me to see that, though there are structual drawbacks inevitable to a building which was erected many years ago, the patients are excellently provided for in medical and surgical wards that are large, airy, and as well arranged as possible. Improvements have recently been effected in ventilation, there is an operating room of good size adequately appointed, and the Roentgen ray apparatus is in frequent use.

#### The Sisters' Quarters.

The covered way from the hospital to the sisters' quarters is not yet completed. It will be a great convenience in bad weather, but in the bright sunshine the want of it is not felt. A tennis court forms part of a nice little piece of ground which is sacred to the sisters, and their home, opened last June, in which there are 20 bedrooms, also contains handsome and commodious reception and mess rooms. Exposure to the east winds which blow across the water is the only drawback to a healthy and pleasant situation. First of all I inquired about the presentation of the new badges and new warrants.

#### The Queen to Visit Haslar.

"They have only been presented at Plymouth and Dartmouth as yet," Miss Mackay replied, "because we believe the Queen intends to present them herself. We are hoping that she will come to Haslar before long. It is a great satisfaction to us to know that Her Majesty takes such a personal interest in the Naval Nursing Service. The badge, as probably you know, consists of an Imperial crown, a gold anchor with the Queen's monogram in red crossed over, and

THE NEW BADGE.

a red cross on a white ground encircled by a gold ring. The new commissions are signed by the Queen herself."

"Of course, the Naval Service is in many respects different from the Army Service?"

"There are a good many differences. One is that each naval hospital has its own arrangements. The arrangements at Haslar are not necessarily the same as those at Plymouth or Chatham."

"But do not the general regulations apply to all the hospitals?"

"Yes, certainly they do. They are issued by the Admiralty. The Director-General of the Medical Department of the Navy, Northumberland Avenue, London, makes all the appointments, and applications for admission to the service should be addressed to him."

#### The Altered Regulations.

"Have the regulations been altered lately?"

"About a year ago they were somewhat altered. No alteration was made in respect to the primary condition, and, whatever may happen in the future, that will remain the

### QUEEN ALEXANDRA'S NAVAL NURSING SERVICE.—*Continued.*

same. It is indispensable that candidates should have gone through three years' training at some large hospital to which a medical school is attached. The alterations were chiefly as to age and pensions."

"How have they been altered as to age?"

"Formerly, the age of admission was between 25 and 38. It is now between 25 and 30. The age of retirement was 60, now it is 50. The pension is given at 50, or after ten years' service if unfit for duty. Head sisters have to retire at 55 instead of 60."

"What is the number of sisters?"

"Twelve at Haslar, ten at Plymouth, four at Chatham, two at Dartmouth, and five at Malta—33 in all. These include head sisters—one at Haslar, one at Plymouth, one at Chatham."

"You, I think, have been a member of the service from the outset?"

"Yes, I joined at the beginning in 1884. At that time there were no sisters here. We started with nine sisters at Haslar and six at Plymouth. After I had been at Haslar eighteen months I went to Chatham for fifteen months, and thence to Malta, where I was in charge from 1889 to 1892. Every sister has to go to Malta for three years if she is sent."

"Where did you go next?"

"As head sister to Plymouth. I went there on March 16th, 1892, and remained there until the end of 1899. I commenced my duties as head sister at Haslar on January 1st, 1900."

### The Male Probationers.

"That brings me to the system of nursing, and the changes which have occurred since you came here first in 1884."

"The whole system has been changed. At that time odd men from outside did the nursing. That is to say, pensioners and others were taken from the shore into the hospitals without any kind of training."

"And now?"

"The men come as probationers for six months."

"What is the nature of their work during that period?"

"They assist in the wards, are taught nursing practically by the sisters, and attend lectures, with a view to passing an examination in bandaging and dispensing under one of the doctors. The age at which they are admitted is eighteen to twenty-one."

"From what class are they chiefly drawn?"

"They must have a fair education; and, generally speaking, they are a respectable class of men. When they enter they take the rank of a petty officer."

"How many are usually in training?"

"The number varies according to entries. The average is between fifty and sixty."

### The Attendants and Ward Masters.

"What happens to the probationers at the end of the six months' training?"

"They become attendants, and are attached to various wards as nurses."

"Are sailors employed to nurse at all?"

"Never. When the attendants have been in the wards a year or eighteen months they are generally drafted to sea. Each battle-ship takes three or four of what is called the sick-berth staff, probably consisting of the steward in charge, a second class steward and two attendants. As the men go on they pick up their rank, eventually becoming ward-masters."

"Is the position of a ward-master the highest they can reach?"

"Yes. A ward-master is a first-class steward. His duty here is to enforce discipline in the wards, to see that they are clean, and to look after the stores and arrange the details of the duties of the male nursing staff. The head ward-master here, at Plymouth, and at Chatham has warrant rank."

"Am I right in concluding that the attendant in the Naval Service corresponds to the orderly in the Army Service?"

"I believe so. His duties are the duties of a male nurse all through his career, either in hospital or on board ship."

"How many probationers are on duty in each ward?"

"Generally two or three. There are about fourteen beds in each ward. The probationers assist the stewards and attendants attached to the wards."

### The Sisters.

"Where does the sister come in?"

"The nursing is entirely under her superintendence. It is the sister's business to see that the orders of the doctor are carried out by the men."

"Over what number of wards is a sister placed in charge?"

"Four or five during the day and half the hospital during the night. This is necessary because the limited number of sisters does not allow of a different division."

"How many beds are usually occupied?"

"About 700 or 800. There is always a certain proportion of bad cases."

### Hours of Duty.

"Then, one sister takes the medical side and the other the surgical side at night?"

"Yes; night duty is taken in turn by every sister, except the head sister, for a month, no matter whether she joined the service yesterday or has belonged to it from the beginning. Practically the sisters do the whole of the actual nursing, and there is plenty of it to be done."

"Are the hours of duty for the sisters identical in all the hospitals?"

"Each hospital has its own arrangements about hours."

"What time do the sisters start on day duty?"

"Here at 8.30 in the morning. The night sister comes on duty at 8.30 in the evening. The sisters are all off duty every afternoon from 3 to 6, except two, who have two hours in the morning free instead. They are all supposed to have one Sunday off in four, and from 2 to 10 once a fortnight. But they cannot always be spared. The head sister has 40 days' and the sisters 32 days' holiday in the year. We take the days when we like."

"Is there any kind of probationary period on admission to the Naval Nursing Service?"

"Not in the ordinary sense; but the sisters are not confirmed in their appointment until the end of the first year."

### Payment and Promotion.

"What is the rate of payment?"

"The commencing salary of the sisters is £30, and it increases £2 a year up to £50. In addition there are a washing allowance and a provision allowance, and uniform is provided. The salary of the head sister at the various hospitals differs in amount, Haslar coming first."

"When an appointment is made, is the new sister sent here?"

"She may be sent to any of the hospitals."

"How frequently do vacancies arise?"

"Only two or three times a year. There are very few vacancies for head sisters. In 17 years only nine head sisters have been appointed. This means that promotions are slow."

"Are there opportunities for distinction?"

"The sisters rarely have the chance of winning a decoration. Three were attached to the Benin Expedition, and one was awarded the Royal Red Cross."

"Are there any special difficulties to encounter?"

QUEEN ALEXANDRA'S NAVAL NURSING SERVICE.—*Continued.*

"The constant change of the attendants is trying to the sisters. As soon as they are used to a man he is often sent away at short notice."

"Do you anticipate the fact that the Queen has assumed the presidency of the service will result in alterations?"

"Time will show. Whatever changes may be considered desirable in the future, we do not want to have a difference in rank among the sisters. At present all rank alike and perform the same duties, and every member must be of good social position. We are regarded as officers, and rank immediately below surgeons. Now that the Queen has taken us under her charge, we hope that she will show she is no less interested in the naval, than in the army, service."

### Nursing in the Burgher Concentration Camps.

BY K. B. BRERETON, A.N.S.R., A MEMBER OF THE LADIES' COMMISSION.

SOON after concentration camps were started the need for trained nurses and proper accommodation for the sick became very evident. The epidemic of measles began to make itself felt, followed in so many cases by broncho-pneumonia and other complications requiring skilled nursing that when, just seven months after camps were first formed, the Ladies' Commission commenced their tour of inspection, they found each camp with its own hospital quarters, and—with one exception only—with its trained nurse or nurses.

### HIDING SICK CHILDREN.

At first the dislike, the terror of going into hospital, evinced by the Boer women for themselves and their children was an almost insurmountable difficulty for the superintendents and doctors to cope with. The people refused to send for the doctor, hid their sick children when he visited the tents, and in some instances even carried them out of the wards when, in order to save their lives, it had been necessary to order them into hospital. Time after time cases of infectious diseases were concealed, and were a source of danger to every occupant of the tents; and yet this attitude of the people was more to be deplored than wondered at, living as so many had previously done in isolated districts where neither birth nor death certificate was required, scarcely ever seeing a doctor, and certainly, except in the few large towns, never hearing of a place set apart for sick people. It was natural that in their ignorance they should resent and fear the idea of their children and friends going to strangers in their sickness; still, on the other hand, to realise the question from their point of view is to understand to some extent the tact, patience and firmness necessary on the part of the officials to overcome these prejudices and teach them what was best for themselves and their children.

### THE HOSPITAL.

There has always been an evident desire on the part of the authorities to make the hospital the most comfortable and perhaps the smartest place in the camp; the enclosure was either railed off or marked out with whitened stones after the manner of the military hospitals. The tents supplied for the use of the patients were either marquees or the best kind of Indian pattern called "E. P." In some camps wards were built of sun-dried brick, roofed with corrugated iron; in others the regular wood and iron huts were erected, and these of course were the best. In the tents the floors were either hardened earth or covered with black sail-cloth. The beds, the same as those supplied to the Army hospitals, were sometimes of the stretcher pattern, or, more generally, iron frames and wire-woven mattresses, and pillows, sheets, and blankets as supplied for our sick soldiers. In those parts of the Colonies where transport and wood were a great difficulty, a table in the middle of the tent would practically complete the equipment, but it was a pleasure to see over and over again that, when the regulation Army lockers were not forthcoming, cleverly-made cupboards and bedside tables had been contrived from old packing-cases, and it was still more touching to observe the variety of ways in which the nursing sister would brighten up the tent or ward with some pretty cloth, plants, or flowers; just these womanly touches which, as we know, make all the difference in our wards at home. There was generally in some central position a sun-dried brick kitchen and pantry combined, with cooking and Soyer stove, the latter generally used as well for broth making for the camp. There was also a place set apart for the hospital laundry, and more or less successful attempts for boiling or otherwise disinfecting linen and destroying infected stools. The dispensary, often located in a marquee, was fitted with shelves and cupboards ingeniously contrived out of old medicine cases, and a counter run up from the same useful material. In most camps the supply of drugs was excellent and the medical comforts, often issued from the dispensary, generally included champagne, brandy, port wine, mazeine, cornflour, arrowroot, Bovril, Brand's essence, jelly, tea, milk, butter, jam, Quaker oats, etc.

### THE MEASLES EPIDEMIC.

At the time when the Ladies' Commission visited the camps, the great objection to hospitals was beginning to die out and the authorities were becoming more and more alive to the fact that to keep the camps healthy the sick must be in proper quarters. With the best intention in the world, the nursing of the average Boer women inevitably produces the worst results. Their main ideas on the subject are to admit no fresh air, to allow no washing, or even undressing of the patient, to try, one after another, every remedy suggested by their neighbours as well as the contents of the family medicine box, which is always somehow well supplied; add to this their views on feeding, and the wonder is, not that so many sick died, but that so many recovered. In the measles epidemic, the disease often broke out in every part of a camp simultaneously, making it all the more difficult for the doctors and nurses to meet the sudden increase in their heavy work, or take means to prevent its spread. One camp, exceptionally fortunate in its comparatively low death rate, adopted the plan of moving the whole family into the hospital enclosure as soon as one or two members were attacked. There, although they could not pretend to thoroughly nurse them, the nursing staff could more easily supervise, administer medicine, and provide extra medical comforts and blankets at short notice. In another camp in which the epidemic was raging the P. M. O. started a convalescent camp, to which he moved all patients before they returned to their ordinary tents, giving them extra food and comforts, and he attributed to this plan very good results.

### THE DUTCH PROBATIONERS.

From all accounts the death rate in all concentration camps may now be described as normal, and everywhere the hospital equipment and staff have been increased and many much-needed improvements made for the comfort and welfare of the medical and nursing staff. From the early hospital days Dutch girls living in camp have been employed under the supervision of British trained nurses as probationers. They are paid, and also receive a simple

### NURSING IN THE BURGHER CONCENTRATION CAMPS.—*Continued.*

uniform. Although they have much to unlearn, many have shown themselves good and capable workers, ambitious of becoming "real nurses." I think that at this point a few quotations from a letter received from South Africa in April, and written from one of the largest camps in the Transvaal, may be interesting.

### The Present Position.

After speaking about various departments in the camp, the writer says:—"There is now a hospital for 150 beds, with 113 patients in to-day, and not a single case with a temperature under treatment in tent in camp. The death-rate, as follows, shows wonderful improvement: November, 88; December, 39; January, 1902, 19; February, 15; and of those who died in January a large proportion were cases left over as a kind of legacy from 1901. Many were very old people and others cases of mirasmus and debility. During the first two weeks in January, whilst the hospital was being enlarged, a number of these cases died in camp, but such an occurrence is now almost an impossibility. Sister H. is matron, sister J. is assistant matron; both from G. hospital, as full of work as one could possibly wish. They take great interest in the work, and have a good staff of trained nurses and Dutch probationers under them. There is also a good mess for the doctors and sisters, and likewise one for the probationers on Hospital lines. At first we had the greatest difficulty with the 'Vrows,' they wished to live in the hospital with their sick children and friends, and did not want to let them come in: but they (the staff) have so improved the tone of the institution that the difficulty now is to get them out of the hospital. The visiting hours are reduced to one hour three times a week, except in dangerous illness, when friends can go in as often as they wish. The tents are nicely arranged in lines. There is a Thrush disinfector at work, and all other sanitary arrangements for treating infectious or contagious diseases, so that as a case occurs the tent is struck and with it all the belongings of the patient are at once sterilised." I should not like to say that every hospital can compare with this; at the same time some of the most pleasing memories I have of the concentration camps is the way in which the majority of the officials showed themselves determined to make the best of their surroundings, and, as opportunity offered, to do better.

### The Royal British Nurses' Association and the Midwives Bill.

The "Amendments desired by the Royal British Nurses' Association" to the Midwives Bill were discussed at a meeting of the Council on Wednesday, 14th inst., under the chairmanship of H.R.H. Princess Christian. Mr. Fardon introduced the subject. The first amendment proposed was a verbal one, as follows: On "clause one, subsection one, line 9, after the word 'Act' to leave out all the words as far as the word 'unless' on line 11. Or, as an alternative amendment, to insert a new subsection as follows: "The penalty mentioned in this clause shall not apply to the persons whose names are published in the list maintained by the Royal British Nurses' Association."

The adoption of the first amendment, Mr. Fardon said, would leave the clause as it stood when the Bill was read a second time. The adoption of the second would protect the members of the Association in the privileges they already enjoyed under their Charter of Incorporation, and obviate the liability to the penalty mentioned in the clause of those nurses whose names appeared in the special list. It might be obviated by registration, but that would imply that they were engaged in practice as midwives, which a large majority did not desire, besides subjecting them to the regulations contained in Clause 10, which might be very difficult for them to carry out.

The second amendment was, from Clause 2, page 2, to leave out lines four and five. The effect would be to throw upon the Central Midwives' Board the responsibility of examining into the character, antecedents, and fitness of candidates. Mr. Fardon then went over the grounds for this amendment, his main point being that the L.O.S. was merely an examining body, and that its certificate was no guarantee of training; he contended that the society should be put upon the same level as others.

Dr. Galton said he was in cordial agreement with the first amendment, but with regard to the second he was not at all sure of the ground. The Bill applied to women who were practising, at fees which he hoped none of the Association's nurses would take, without any control whatever. He thought it was a mistake to connect the Association's nurses with those willing helps who were necessary to the poor, and he held that the L.O.S. had been doing a good work. He did not believe such an amendment would have any chance of acceptance. The effect of registration would at first be just what it had been in the case of medical men—some undesirable persons must be among the number. Women holding the L.O.S. certificate had a higher claim than others to practise among poor people. One word as to the action taken a fortnight ago. He would be sorry to think that there should be an impression that the amendment then put was carried by so small a majority; he did not think members should refrain from voting.

Dr. W. S. A. Griffith said he hoped that no decision would be come to in a small meeting and without specific discussion. He was opposed to the second amendment; if it were passed, the Association would find itself a tool in the hands of those who were opposing registration; and possibly the whole question of registration might be wrecked again. The L.O.S. stepped in 20 years ago, when there was no other body to take the matter up. He was not speaking as a representative of that body, but simply as one of the surgeons at Queen Charlotte's Hospital. When once the Central Midwives' Board was formed, the L.O.S. would be most happy to hand things over to it. Those who thought the Board would have nothing to do were ignorant of the whole matter; he only hoped the Board would receive some payment, for it would have to work very hard. He sincerely trusted that the amendments would not be passed; they should be put on the agenda and circulated to all the members, and then an opinion could be arrived at. He thought it most highly improbable that any special exemptions would be made when once registration was settled.

Mrs. Latter said she was most cordially in agreement with Dr. Griffith; and it was ultimately decided to discuss the matter in conference.

### Presentations.

Rotherham Hospital.—The late matron, Miss Sanders, was the recipient of many handsome and valuable presents upon the occasion of her marriage, which included an oak secretaire from the Board of Guardians, a silver-mounted dressing-bag from the chairman, silver sweet-dishes from the doctors, a silver cake-basket from the past and present nursing staff, a tea service and jam-dish from the servants.

Sarsden and Churchill District Nursing Association.—Miss Elizabeth Broomhead, who has for several years been nurse at Sarsden and Churchill District, has left for the purpose of taking up nursing on her own account. Prior to her departure the residents presented her with a valuable nursing-bag with accessories, also an umbrella, in acknowledgment of her services whilst she has been labouring amongst them.

### Appointments.

[No charge is made for announcements under this head, and we are always glad to receive, and publish, appointments. But it is essential that in all cases the school of training should be given.]

BEDFORD ISOLATION HOSPITAL.—Miss Helena Berryman has been appointed matron. She was trained at Richmond Hospital, Surrey, and has since been charge nurse at Wrexham and Ramsgate Fever Hospitals. She has also done eleven years' private nursing.

BELPER WORKHOUSE HOSPITAL.—Miss Catherine Edith Claredge has been appointed staff nurse. She was trained at Chorlton Union Hospital, and has since been nurse at Sherburn Hospital, Durham, and at the Hull and East Riding Withernsea Convalescent Home.

BROMSGROVE DISTRICT AND REDDITCH ISOLATION HOSPITAL. — Miss Theresa Russell has been appointed matron. She was trained at the Nightingale School, St. Thomas's Hospital, London, and has since been sister of the operating theatre, nurse in the isolation block, and sister in the male surgical ward of the same institution.

CITY HOSPITAL, FAZAKERLEY, LIVERPOOL.—Miss Madeleine McArdle has been appointed deputy matron. She was trained for three years at Birkenhead Infirmary, and has since been charge nurse at the City Hospital, Liverpool, and the Park Hospital, Lewisham, London.

COUNTY CAVAN INFIRMARY.—Miss Katie Sheridan has been appointed matron. She was trained at the North Charitable Infirmary, and the District Union Hospital, Cork.

FULWOOD WORKHOUSE INFIRMARY, PRESTON. — Miss Ann Jane Atherton has been appointed superintendent nurse. She was trained at Crumpsall Workhouse Infirmary, and has since been superintendent of infectious wards at Fulwood Workhouse.

HENDON SICK ASYLUM.—Miss Emily Susannah Ludwig has been appointed charge nurse. She was trained at the City of London Infirmary, Bow Road, E., where she was afterwards staff nurse, and she has also been trained as a midwife at Walthamstow.

INSTITUTION FOR TRAINED NURSES, DOVER.—Miss M. Turner has been appointed lady superintendent. She was trained at the West Kent General Hospital, Maidstone, and was afterwards on the private staff of that hospital. In 1893 she was appointed lady superintendent of the County of Cornwall Trained Nurses' Home, and for the last six and a half years has been matron of Repton School Sanatorium.

MONTGOMERYSHIRE INFIRMARY, NEWTOWN.—Miss Margaret Storey has been appointed matron. She was trained at Leeds General Infirmary, where she was afterwards theatre operation and ward sister. Subsequently she has been matron of Fleetwood Cottage Hospital and matron of the Palmer Memorial Hospital, Jarrow-on-Tyne.

ROYAL INFIRMARY, HULL.—Miss Edith Gregory has been appointed home sister. She was trained at St. Bartholomew's Hospital, London, and was afterwards attached to the private nursing staff. She has since been theatre sister at the Liverpool Hospital for Women, and ward sister and night superintendent at the Norfolk and Norwich Hospital.

ST. ANNE'S HOME, HERNE BAY.—Miss Rosa Bashford has been appointed charge nurse. She was trained for three years at the Alexandra Hospital for Children with Hip Disease, London, and was afterwards nurse in the same institution for six years.

ST. OLAVE'S INFIRMARY, ROTHERHITHE.—Miss. S. Angus has been appointed charge nurse. She was trained at St. Pancras Infirmary, London, and has since been charge nurse at Grove Hospital, Tooting, private nurse at Swansea and South Wales Nursing Institute, and district nurse at Swansea.

SOUTHAMPTON ISOLATION HOSPITAL.—Miss Maud Lizzie Barnes has been appointed charge nurse. She was trained at St. Saviour's Infirmary, East Dulwich, and has since been nurse under the Metropolitan Asylums Board at the South-Eastern Hospital, and at Cuddington Isolation Hospital.

STROUD GENERAL HOSPITAL.—Miss Ellen Overbury has been appointed sister of women's ward. She was trained for three years at Guy's Hospital, London, and has since been charge nurse at the Western Fever Hospital.

THOMPSON MEMORIAL HOME, LEITRIM.—Miss Currie has been appointed sister. She was trained at the Royal Southern Hospital, Liverpool, and has since been sister at the Home for Incurables, Liverpool, for four years, taking matron's holiday duty.

WATH, SWINTON, GREASBOROUGH, AND NORTH ROTHERHAM JOINT HOSPITAL.—Miss Edith Harris has been appointed charge nurse. She was trained at the Bristol Workhouse Infirmary for three years, and has since been charge nurse at the Ham Green Fever Hospital, Bristol. Miss Harris holds the L.O.S. certificate.—Miss A. Carnall, Miss E. Mee, Miss E. Luff, and Miss L. Hutson, have been appointed assistant nurses. Miss Carnall was trained in fever at the City Hospital, Lodge Road, Birmingham; Miss Mee at the Uxbridge Joint Hospital; Miss Luff at the Isolation Hospital, Surbiton; Miss Hutson at the Isolation Hospital, Blackpool.

### Death in Our Ranks.

WE regret to hear of the death of Miss Frances Holcroft, a member of the Army Nursing Service Reserve, who died at Belsize Cottage, Belsize Lane, Hampstead, last week.

### TRAVEL NOTES AND QUERIES.

To BERWICK-ON-TWEED FROM CHESTER (Gothic).—It is quite a direct line, but I cannot give you the cost of it. The railways only publish prices from London, but you have only to ask at your own station. Go to the Red Lion Hotel and look for rooms; we do not give the name of lodgings; I should think they would be reasonable.

LUCERNE FOR A HOLIDAY (Alnwick).—Second return *viâ* Brussels and Strassburg is £5 7s. 6d. Good accommodation can be had for 7 francs per day. Reckon £2 per week. It would probably be considerably cheaper to go with a party, but this disadvantage is that the time is usually very short. Write to Dr. Lunn, 32 Piccadilly Circus, W., and ask what Swiss tours he has on at the time you wish to go, and write to me again if I can heip you. Tell him who recommended you to him; you are likely to receive more attention.

ISLE OF MAN (Francesca).—There is no charge unless you want an answer by post, when we ask for 2s. 6d. for our Convalescent Fund. I can help you better when I know more. The Isle of Man is a big place; when you tell me something of where you wish to go in it I will send you addresses. If you mean to go into a boarding-house you must stay all the time in one place, as proprietors do not like to take guests for less than several days. Roughly speaking, I think you might manage a week in *one* place for 30s. to 35s., exclusive of journeys. The roads are excellent for cycling. You would be able to form some idea of the localities you would prefer if you wrote to the office and asked for my article on the Isle of Man, published June 30th, 1900. Expense from Dublin you will learn at your own station. Write me again more fully.

To BRITTANY CYCLING (Eleanor).—If you follow this route you will do the month for £10 each or a very trifling sum over, but you *must* go by sea to St. Malo and return the same way, second return £2 1s. 2d., a little less if you go third from London to Southampton. Second on the boat is unexceptionable. Start on a Friday, arrive Saturday morning. Go to Dol and see the market, land and rest, then on to Mont St. Michel, 40 miles altogether. Put up at Hôtel Poulard Aîné. On Monday train to Dinan to save time and fatigue. From there cycle 38 miles to St. Brieuc and put up at the Croix Blanche. Next day cycle to Paimpol; sleep at Hôtel Michel. Then cycle round the coast to Lannion, where you must sleep. Then cycle to Morlaix, only 20 miles. At Morlaix go to the Hôtel de l'Europe. Always ask for rooms on the third floor. If you do not spend more than 7 francs per day you can just manage within the £10, but changing residence so always means expense. A much cheaper way would be to see Mont St. Michel and then go to such a place as Tréquin, north of Guingamp, where you could be in the convent of Les Sœurs de la Croix, or at Guingamp also Les Sœurs de la Croix. I think you would get good accommodation for 5 or 6 francs per day. You could then visit much of the surrounding country. Send on your small luggage by *post*; it is the safest way. No, there is nothing to pay. You will find information on the parts you want in THE HOSPITAL for 6th and 13th May, 1899.

### Everybody's Opinion.

[Correspondence on all subjects is invited, but we cannot in any way be responsible for the opinions expressed by our correspondents. No communication can be entertained if the name and address of the correspondent are not given as a guarantee of good faith, but not necessarily for publication. All correspondents should write on one side of the paper only.]

### NURSING AT WHITBY ISOLATION HOSPITAL.

"Sister" writes under date of May 13th: I hope by giving publicity to these facts to draw the attention of the sanitary authorities of Whitby to the grave mistakes they are making in the management of their Isolation Hospital situated in the parish of Stainsacre. Last week there were 17 patients (diphtheria) in 13 beds. There are no sanitary arrangements whatever; no W.C.; the contents of bed-pans, etc., are thrown in an open hole in a field 15 or 20 yards from the ward. There is one sink in the kitchen where everything is washed, including the nurses' cups and plates, and also the ward utensils etc. There is no bath-room. When a patient leaves, a tub is taken into the ward, the patient is bathed and dressed there and sent straight home from the ward. The only means of dininfecting clothes, bedding, etc., is in an old cab, in which a little sulphur is burnt. The nurses have no room of any kind. They either sleep in the diphtheria ward or in a small passage near the kitchen. This is for the night nurse as well as for the day nurse. This spring a nurse developed diphtheria and was ill for a considerable time. The matron's husband is the porter; whether she is trained or not I do not know. The authorities wonder why diphtheria still rages in the town. I hope that someone will help them to understand the reason "why."

### CAMBRIDGE SANATORIUM.

"A Former Matron" writes: I noticed in The Hospital a few weeks ago a paragraph respecting charges which had been brought against the management of the Cambridge Infectious Diseases Hospital. I know nothing of the matron or her staff, whether capable or otherwise; but I do know the great difficulties attending the position of matron of the Cambridge Sanatorium. As far back as 1894 the domestic and permanent nursing staff was insufficient, and the matron suggested alterations which would have been both efficient and economical. The following is the manner in which her suggestions were treated: At a meeting of the sanatorium committee the following recommendation of the sub-committee was adopted: "Having heard the views of the matron, and of Nurse ——, and the opinion of the M.O.H., the sub-committee are of opinion that no alteration is needed in the present staff of nurses, and the appointment of a ward-maid is unnecessary." I may say that the views of the M.O.H. were entirely in support of the matron, but fancy any enlightened committee consulting a nurse in the matter. Needless to say, when the matron was made acquainted with the resolution, her resignation was immediately lodged with the town clerk. On another occasion the chairman of the committee was of opinion that the Medical Officer of Health who was medical superintendent of the hospital, should not have used a certain block for a certain disease, and wanted the matron to undertake to report any similar proceeding in the future to himself or the town clerk; the matron naturally refused to report any legitimate action of her superior officer to anyone, chairman or otherwise, and thereby incurred the grave displeasure of the chair. The matron is not invested with the authority which she ought to possess if her management is to be efficient, and so long as the committee insist upon retaining so much in their own hands, they need not hope for a peaceful administration. The paragraph mentioned above did not appear to me to be in sympathy with the matron, and though she is totally unknown to me, I felt that I should like to state my experience of the many difficulties attending the position of matron of the Cambridge Sanatorium.

### MALE NURSES.

"Male Nurse" writes: As a male nurse, I have read the various comments regarding male nurses in private work and institutions, and W. Gutteridge, whilst upholding "male nurses," brought them all up to an equal with female nurses. Now that is where he spoiled the effect of his letter. Some there are, I admit, who having had medical, surgical, and mental training, might be placed equally on a level with the best of female nurses, but a nurse who has had only a slight training in medical nursing, even though he may be an excellent mental nurse, cannot be placed on a footing with the trained female nurse. They are both efficient, but each in their own capacity. There are male nurses who have had medical, surgical, and mental training, but the large majority have not. With regard to general hospital training, it is a pity that there is not more scope for male nurses to enter the same and obtain a full training. As regards character, male nurses are under a ban; they are not respected as they might be; there are, of course, "black sheep" in every profession, but the majority of male nurses are men of good education and social position as far as it goes. Were they respected more by the public, and their merits recognised, the action would be much more appreciated by male nurses than it is at present.

### THE POSITION OF PRIVATE NURSES.

"An Interested Spectator" writes: I should like to say a few words regarding the position of private nurses, as I frequently read The Hospital, and have been interested in the various opinions expressed on the subject, besides which, having relatives among the maligned band, I hear a good deal, from time to time, as to the way in which they are treated at different houses. It is very much regretted that many nurses, although skilful in their work, are lacking in womanly sympathy, but does it ever occur to those employing nurses that, although skill can be purchased, it is utterly impossible to buy real genuine sympathy that must arise spontaneously from the nurse's inner feelings, and if private nurses are treated in such a manner as to make them grow hard and sceptical, is it any wonder if they lose their fineness of sympathy? I have heard a nurse say, "In cases where I am made one of the family, there is no sacrifice which I would not make, either for the patient or her friends, as readily as I would for a loved one of my very own; but in cases where I am treated merely as a necessary encumbrance (which I am happy to say does not often occur), although not losing my interest in my patient as a case, and performing my duties no less faithfully, I cannot feel the same heartfelt sympathy with all around as I do under other circumstances." I think myself that that is only natural, and if people feel the need of genuine sympathy from their nurses they should not treat them in a manner calculated to dull their sympathies. As regards a nurse's social position in a household, I take it that she enters in a professional capacity, and there ought to be no more question of asking her to take her meals with servants of any description than there would be if it were necessary for a doctor to have a meal in the house. There are many unrefined nurses who, as a "Sister of a London Hospital" puts it, are fond of retailing bits of gossip to servants, but if those nurses were put on a professional footing, and not thrown so much in the way of servants, they would not have the opportunity of gossiping with them. Surely when nurses spend the greater part of their time in "ministering," it is not too much for them to expect to be "ministered to" a little during their time away from the sick-room, especially in houses where there are servants and no lack of any comfort for the members of the household. There are some doctors to whom one could not exactly apply the title of gentleman, but no one would think of asking them to have a meal with servants if there were any reason why they should be resident in the house. To quote once more the "Sister of a London Hospital," private nurses are only "birds of passage," and where people are really nice they are so thankful to have a nurse who can alleviate the sufferings of their "loved one" as they are unable to do, that they gladly make her one of themselves for the time being, and do everything that they can to make her comfortable during her time out of the sick-room, realising what it would be for themselves to be shut in for so many hours, although the nurse gets her remuneration for her work.

### Echoes from the Outside World.

### The Coronation.

The preparations for the Coronation Ball at the Crystal Palace on July 2nd, in aid of King Edward's Hospital Fund, are being made with the approval of His Majesty under the presidency of Mrs. Arthur Paget. Twelve thousand square feet of the floor of the central transept will be laid with parquet flooring, and will be the largest area ever laid down for dancing. In the south transept four great drawing-rooms will be laid out, and from these a huge bank of flowers and plants from the Palace greenhouses will rise to the Royal box. The furnishing of two of these in Louis XVI. and in English style has been given as a special donation to the Fund. Beneath the balconies on either side of the drawing-rooms will be two gardens, one in English style, with a green winding-path and flower-beds, the other Italian, with statuary, palms, and a fountain-pond. The ball will commence at 10 o'clock, and special trains for the return journey will run until well into the morning.

The guests whom the Prince and Princess of Wales have invited to Marlborough House for the Coronation include no fewer than 2,400 children—1,200 on the 26th and 1,200 on the 27th of June. Stands are to be erected so that they may all get a good view of the procession, and afterwards dinner is to be served in the garden. Amongst those who have already received invitations are the children from the Merchant Seamen's Orphan Asylum, Snaresbrook, and those from the Foundling Hospital, which was originally founded by a master mariner. So that the Prince has evidently especially remembered the days when as a youth he learnt the art of navigation. It has been arranged that not only shall each child see the procession and dine, but shall also receive a souvenir of the event in the shape of a mug upon which will be portraits of the King and Queen, the Prince and Princess of Wales, and the date of the Coronation.

In addition to the exhibition of the Prince of Wales's presents which has just opened at the Imperial Institute, there is another collection of an interesting character to be seen at the British Museum just now in connection with the Coronation. It is in the King's Library, and it includes a series of manuscripts, printed books, drawings, and medals in illustration of coronations from the earliest times. The first is the volume of Gospels upon which tradition says the Kings of England formerly took the coronation oath. Anyhow, it dates back to the time of Athelstan, A.D. 940. Then, amongst much worthy of notice, there is the pledge of Ethelred, the form of words having been ordered by St. Dunstan; a proclamation made by Richard III. before his coronation; a few old tickets to admit to previous coronations, and the medals dating back to Edward VI. To those who are fond of antiquities this collection will be found exceptionally interesting.

### Foreign.

On Saturday the young King of Spain attained his majority. Alfonso XIII. is only 16 years of age, but that is the age of accession prescribed by the Constitution. It is a remarkable fact that this King was born a King, and therefore enjoys one unique distinction amongst the monarchs of history. At the present moment he is described as tall and slender, graceful in movement in spite of the length and looseness of his limbs, and he is said to have both the mobile features and very much of the charm of manners which made Alfonso XII. so dear to his friends. He has been educated with great care, and has proved an apt pupil. He has been taught to fence, is a fair shot, and rides with pluck and judgment. Of course he has been instructed in military drilling and exercises, and shows signs of becoming a keen soldier. The ceremonial in the Plaza de las Cortes was of a brilliant character, but an extraordinary incident occurred as the King left the Escurial Palace. A man approached the Royal carriage, and throwing his hat and a paper into it declared that he wanted to marry the Infanta Maria Theresa. He was promptly taken into custody, and it is said that he is a maniac. But the report spread among the crowd that the life of the Sovereign had been attempted, and created immense excitement. There seems also to have been some cause for uneasiness, for several suspected Anarchists have been arrested, and on one of them dynamite cartridges were found. The King was received with great enthusiasm in the Cortes, and took the oath of fidelity as follows:—"I swear by God upon the Holy Bible to maintain the Constitution and the laws. If so I do, may God reward me. If I do not, may He call me to account." A farewell letter from Queen Maria Christina to Señor Sagasta on her retirement from the Regency has been issued, and also a protest from Don Carlos against King Alfonso's accession. He asserts his right to the throne, and describes the young King as "an unlawful intruder."

### Colonial.

It was announced on Thursday last week that Lord Hopetoun had resigned the post of Governor-General of the Australian Commonwealth; and it has since transpired that this step is due to the refusal of the Federal Parliament to grant him additional annual allowance pending the establishment of a federal capital. Lord Hopetoun does not complain of the past, but he says that he cannot afford the continued drain on his resources. It is understood that since his appointment he has spent £25,000 out of his private purse. Mr. Chamberlain has signified his regret at the refusal of the Australasian Parliament to grant the increase, and he has intimated that he could not refuse to acquiesce in the request of his Excellency who asks to be relieved of his post after the Coronation. Lord Hopetoun is very popular at the Antipodes. The State Premiers and their colleagues, in conference at Sydney, have sent him a telegram expressing their deep and sincere regret at the announcement of his retirement, to which they added a hope that a way may yet be found to enable him to retain the position for some time to come. They affirm that by his "ability and courtesy he has won the esteem of all classes of the Commonwealth."

### Whitsuntide Disasters.

It is some years since England has experienced such unsatisfactory weather at Whitsuntide as in 1902. In London cold and wet conditions prevailed, with now and then delusive bursts of sunshine; consequently the places of amusement were crowded, and the excursions into the country neglected. In Ireland a sad boating catastrophe resulted from the boisterous weather, thirteen people—nine tourists and four boatmen—having been drowned in the Lower Lake at Killarney. The boat overturned, and not a single person on board was saved. The same evening a tornado swept over the town of Goliad, Texas, U.S., destroying three churches and 100 houses, and killing or injuring 200 people. Trains with physicians, nurses, and State militia hurried to the scene of the disaster, and the court house and private houses have had to be utilised as hospitals. Early on Monday morning a serious explosion occurred at the Fraterville coal mine at Tennessee, also in the United States. Three hundred men were in the pit at the time of the catastrophe, and only one escaped. On the Continent, sightseers were obliged in many places to remain in their hotels, owing to the inclemency of the season; between Italy and Switzerland in particular the passes being impassable, with five to eight yards deep of snow. The mountain railways had to suspend work.

### For Reading to the Sick.

TRINITY SUNDAY.

The Third Day-hour abounded
With Grace, that we might know
The Source of Blessing, Threefold,
Whence Benedictions flow:

And now, on this glad morning,
The best and chief of Days—
Sole Trinity, we bless Thee
In Hymns of grateful praise.—*Dix.*

The doctrine of the Trinity is not discoverable by reason, but agreeable to reason. It corresponds to upward-soaring trains of thought which reason itself originates, but is not able to bring to a conclusion. For the reasons which lead us to believe in God at all, lead us to think of Him as an eternal and spiritual being. Now the life of spirit, the highest life we know, is made up of the action of will and reason and love. In God then, we imagine, is a perfect and eternal life, of will and reason and love.—*Bishop Gore.*

Thee, One in Nature, One in Throne,
Eternal Comforter, we own,
With God the Father and the Son,
The ever-blessed Three in One.

*Dix.*

As in the human spirit, so, too, in the outward world of nature, there aie certain indications and reflections of the Trinity. This truth is not only revealed in Scripture . . . but it is, so to speak, omnipresent throughout the world. We constantly see one *life* in various members; in each one it acts in a special manner, yet in all it is one and the same. In one *sun* we see light and warmth as different, and yet intermingling and co-operating forces. We have *space* divided into three dimensions of length, breadth, and height; *time*, similarly, into past, present, and future.—*Christlieb.*

Most High and Holy Trinity!
Who of Thy mercy mild
Hast form'd me here in Time, to be
Thy Image and Thy child:
O let me love Thee day and night
With all my soul, with all my might;
O come Thyself, my soul prepare,
And make Thy dwelling there!

Most High and Holy Trinity!
Draw me away from hence,
And fix upon Eternity
All powers of soul and sense!
Make me at one within; at one
With Thee on earth; when life is done,
Take me to dwell in light with Thee,
Most High and Holy Trinity!

*Anon.*

Therefore, if we would rise to the higher life, of looking for and hasting unto the coming of the Day of God, we must never allow ourselves, when meditating or speaking on such a subject, to be long without flying back as it were into the over-shadowing Presence, and at least saying in our hearts: "The Grace of our Lord Jesus Christ, and the Love of God the Father, and the Fellowship of the Holy Ghost, be with me!"—*Bishop Wilkinson.*

### Notes and Queries.

**The Editor is always willing to answer in this column, without any fee, all reasonable questions, as soon as possible.**

**But the following rules must be carefully observed:—**

**1. Every communication must be accompanied by the name and address of the writer.**

**2. The question must always bear upon nursing, directly or indirectly.**

**If an answer is required by letter a fee of half-a-crown must be enclosed with the note containing the inquiry, and we cannot undertake to forward letters addressed to correspondents making inquiries. It is therefore requested that our readers will not enclose either a stamp or a stamped envelope.**

*Artificial Foot.*

(64) A girl in my parish has had her foot amputated, and an artificial foot will have to be procured. Do you know of any society which would afford help, as her people are poor?—*Parish Nurse.*

Perhaps the Surgical Aid Society, Salisbury Square, Fleet Street, E.C., can help you.

*Home.*

(65) Can you tell me where a boy of twelve suffering from phthisis, and another of five suffering from incipient phthisis can be received for treatment? I should like also to know where a boy of three who has chorea and is slightly imbecile could be received permanently.—"*Sister.*"

Children are received for treatment at many of the special hospitals for consumption. The Royal National Hospital for Consumption, Ventnor, Isle of Wight; the Mount Vernon Hospital for Consumption, Hampstead; the Hospital for Consumption, Brompton, etc. You give so few particulars that it is impossible to select an institution. The third case might be suitable for one of the children's hospitals, but not "permanently."

Can you tell me of a free home for a child who is an idiot and who has fits? The mother is a widow without means.—*E. M.*

We are afraid that the child must be sent to the workhouse.

Would you be good enough to tell me if there is any hospital where a little imbecile boy of six could be received? It is an exceptionally sad case, as the child is speechless and unable to walk. The parents are poor but very respectable people, who could pay 4s. a week for the child's maintenance.—*F. M.*

Apply to the secretaries of the Earlswood Asylum for Idiots and Imbeciles (office), 36 King William Street, London, E.C.; the Royal Albert Asylum and Training Institution for the Feeble-minded of the Northern Counties, Lancaster; and to the Schools for Imbecile Children, Darenth, Dartford, Kent.

*Dispensing.*

(66) Would you kindly let me know where I can have lessons in dispensing in Newcastle-on-Tyne or neighbourhood?—*Nurse.*

You might make arrangements with a chemist to give you private lessons in dispensing if there are not some public classes in connection with the College of Science which you could attend.

Can you tell me of any dispensary which would take a lady probationer in dispensing, and where I could get the cheapest training?—*G. A.*

Apply to the Secretary, the London (Royal Free Hospital) School of Medicine for Women, 8 Hunter Street, Brunswick Square, W.C.

*Fee.*

(67) Will you kindly inform me what fee, if any, is usually charged in case of a patient dying in a nursing home, and the body remaining two nights over the period for which payment has been made?—*F. T. W.*

An arrangement should have been made at the time of the death. Failing that, the payment should not be less than if the patient had been alive for the two nights.

*Safety-pins.*

(68) Will you kindly tell me what I can apply to safety-pins in order to make them pass easily through stiff binders and which will prevent the pin rusting?—*Nurse B.*

Buy good pins. You can get a good steel one at a reasonable price. A touch of vaseline will greatly facilitate their introduction.

**Standard Books of Reference.**

**"The Nursing Profession: How and Where to Train." 2s. net; post free 2s. 4d.**
**"Burdett's Official Nursing Directory." 3s. net; post free, 3s. 4d.**
**"Burdett's Hospitals and Charities." 5s.**
**"The Nurses' Dictionary of Medical Terms." 2s.**
**"Burdett's Series of Nursing Text-Books." 1s. each.**
**"A Handbook for Nurses." (Illustrated). 5s.**
**"Nursing: Its Theory and Practice." New Edition. 3s. 6d.**
**"Helps in Sickness and to Health." Fifteenth Thousand. 5s.**
**"The Physiological Feeding of Infants." 1s.**
**"The Physiological Nursery Chart." 1s.; post free, 1s. 3d.**
**"Hospital Expenditure: The Commissariat." 2s. 6d.**
**All these are published by the Scientific Press, Ltd., and may be obtained through any bookseller or direct from the publishers, 28 and 29 Southampton Street, London, W.C.**

## Figures and Tables

**Figure f1:**